# Phenotypes, Developmental Basis, and Genetics of Pierre Robin Complex

**DOI:** 10.3390/jdb8040030

**Published:** 2020-12-05

**Authors:** Susan M. Motch Perrine, Meng Wu, Greg Holmes, Bryan C. Bjork, Ethylin Wang Jabs, Joan T. Richtsmeier

**Affiliations:** 1 Department of Anthropology, The Pennsylvania State University, University Park, PA 16802, USA; 2 Department of Genetics and Genomic Sciences, Icahn School of Medicine at Mount Sinai, New York, NY 10029, USA; meng.wu@mssm.edu (M.W.); gregory.holmes@mssm.edu (G.H.); ethylin.jabs@mssm.edu (E.W.J.); 3 Department of Biochemistry and Molecular Genetics, Chicago College of Osteopathic Medicine, Midwestern University, Downers Grove, IL 60515, USA; bbjork@midwestern.edu

**Keywords:** mandible, micrognathia, nasopharynx, tongue, cleft palate, stickler, Treacher Collins, velocardiofacial syndrome

## Abstract

The phenotype currently accepted as Pierre Robin syndrome/sequence/anomalad/complex (PR) is characterized by mandibular dysmorphology, glossoptosis, respiratory obstruction, and in some cases, cleft palate. A causative sequence of developmental events is hypothesized for PR, but few clear causal relationships between discovered genetic variants, dysregulated gene expression, precise cellular processes, pathogenesis, and PR-associated anomalies are documented. This review presents the current understanding of PR phenotypes, the proposed pathogenetic processes underlying them, select genes associated with PR, and available animal models that could be used to better understand the genetic basis and phenotypic variation of PR.

## 1. Introduction

Pierre Robin is an ill-defined disorder with specific mandibulofacial involvement that continues to defy a consistent definition. Since being named for the physician who provided an early description [1,2], it was variously defined as a set of anomalies that can include micro- or retrognathia, glossoptosis, respiratory obstruction, and cleft palate (CP), and termed Pierre Robin syndrome, sequence, anomalad, or complex [3,4,5,6]. Micro- and retrognathia are the most common terms used to describe mandibular phenotypes in mandibulofacial dysostosis, yet the current lack of precision in usage of these terms in diagnoses of mandibular dysmorphology does not critically consider the potentially distinct etiology of these phenotypes and their influence on the possible sequelae of anomalies. Micrognathia describes a mandible that is absolutely reduced in size, indicating that the mandible is primarily affected, while retrognathia refers to a normally sized mandible that is placed posteriorly relative to the upper jaw. Thus, micrognathia and retrognathia, while providing similar facial profiles, are produced by different primary developmental processes, and each may integrate differently with tongue and palatal development. When mandibular dysmorphology occurs with glossoptosis, respiratory obstruction, and in some cases, a CP, the condition is referred to as Pierre Robin (PR), a term we adopt here.

## 2. Historical Perspective

Stomatologist Pierre Robin published an article in 1923 [1] describing a triad of clinical findings in a series of patients, namely, micrognathia, glossoptosis, and obstruction of the upper airways [7]. Following his widely read contribution to the literature on micrognathia in newborns [2], this triad became known as Pierre Robin syndrome by clinicians [3]. Robin considered acquired or congenital glossoptosis as a consequence of a small mandible leading to respiratory problems. These conditions ultimately result in “physical backwardness” in infancy that persists into adulthood. He also introduced the association of these anomalies with CP [2]. Robin linked the respiratory problems in these children to their physical and psychological development, and indicated that infants with severe retrognathia rarely survive beyond 18 months of age [2]. Through the 1960s, clinicians noted that PR generally occurred without other significant birth defects, although the case of a two-month-old male infant with PR and severe bilateral congenital glaucoma indicated ocular involvement in some affected individuals [8]. Natal teeth were associated with one PR patient in a cohort of infants born at Foothills Provincial Hospital in Calgary, Canada, between 1967 and 1984 [9].

The condition was known as Pierre Robin syndrome for nearly 50 years before it was understood that multiple etiologies could underlie the same clinical findings, which did not fit with the prevailing definition of a syndrome: a combination of symptoms resulting from a single cause [10]. In the 1970s, the term Pierre Robin anomalad was introduced [4,5], with the implication that the condition was not a specifically delineated syndrome. Anomalad signifies an etiologically nonspecific complex that can occur as a component of various genetic or teratogenic syndromes of known cause, syndromes of unknown etiology, or as an isolated symptom complex secondary to positional deformation or disruption [11,12]. Anomalad denotes a pattern of morphologic defects that stem from a single, localized, structural anomaly resulting in a cascade of consequent defects [13], so the term implies a sequence of developmental consequences of a primary defect. Hanson and Smith [4] hypothesized the primary pathogenic mechanism of ”Robin anomalad” to be early mandibular hypoplasia with secondary posterior displacement and interposition of the tongue between the closing palatal shelves [4]. The characteristic U-shaped CP of PR individuals [11,14,15], distinct from the more common V-shaped CP, was proposed to have developmental and clinical significance, as well as providing strong support for the proposed etiopathogenetic mechanism involving a small and retropositioned mandible that keeps the tongue high in the nasopharynx, preventing the rotation, medial growth, and fusion of the palatal shelves [4,10]. Cohen presented an extensive review of the conditions in which “Robin malformation complex” can occur along with data useful for diagnoses of patients with cleft lips and/or palates and associated anomalies [16]. By this time, it was recognized that the triad of mandibular hypoplasia, glossoptosis, and a posterior U-shaped CP is a pathogenetically and etiologically heterogeneous condition that can be an isolated defect or one feature of many different syndromes.

Carey et al. [6] used the term Robin sequence in linking the triad to neuromuscular conditions. The term sequence was used with the understanding that there is a temporal succession, and a potential causative pathogenesis, in the order of appearance of the anomalies, namely, primary micrognathia appearing first, followed by glossoptosis and respiratory obstruction, and in some cases, CP [17,18]. The term “sequence” was formally challenged by a comparative analysis of PR and isolated CP patients, but the data examined supported both a sequential genesis initiated by a small mandible and a primary growth disturbance of both the maxilla and mandible [19]. This lack of consensus on whether the condition represents a mechanistic sequence of events resulting from a single primary event (small mandible), a condition of primary growth disturbances of several tissues [19,20,21], or a combination of both processes indicates a need for additional research on the developmental and genetic mechanisms of PR. Such studies could also inform on the etiology of the heterogeneous group of common birth defects, including glossoptosis and CP.

## 3. Epidemiology of PR

The incidence of PR was estimated at between 1 in 8000 to 1 in 14,000 live births in a few epidemiological studies [17,18], and reported as much higher (1:2685 live births) in the East of Scotland region of the United Kingdom [22]. The Dutch birth incidence of PR was estimated to be 1:5600 live births, with a slight female predominance, and was estimated to occur in a third of the CP population, with PR patients having a more severe cleft grade than the general CP population [23]. Another study described PR as having multiple subdivisions [24]. A study based on a population from a large cleft lip and palate clinic in Pretoria, South Africa, differentiated Fairbairn–Robin triad (FRT) from Siebold–Robin sequence (SRS) on the basis of the presence (FRT) or absence (SRS) of CP, with a higher incidence of PR occurring in white males and females relative to other ethnicities surveyed, white females being most commonly affected [24]. Mortality for infants with PR and additional or syndromic malformations was estimated from 1.7% to 11.3%, up to 26% [25,26,27,28,29]. Current literature gives highly variable syndromic frequencies for PR that range from 20–40% [30], while others showed approximately 60% of patients have syndromic features [31]. Overall, the frequency worldwide is unknown, in part because of the lack of consensus about the nature of the condition, and because the occurrence varies with ancestry, geographic location, maternal age, prenatal exposures, and socioeconomic status [32,33].

## 4. Uncertainty of Diagnosis

**Figure 1:**
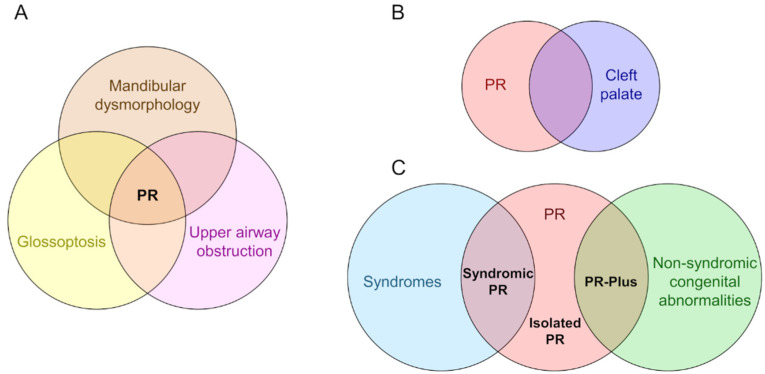
The diagnostic features and categories of Pierre Robin syndrome/sequence/anomalad/complex (PR). (**A**) PR is characterized by a triad of mandibular dysmorphology (micrognathia or retrognathia), glossoptosis, and airway obstruction. (**B**) A U-shaped cleft palate is commonly present in patients with PR, a cleft morphology distinct from the more common V-shaped cleft palate. (**C**) Three diagnostic categories based on whether the PR triad and/or cleft palate appear in isolation or with other anomalies. In syndromic PR, the triad is present as part of a syndrome, appearing coincidentally with Stickler, 22q11.2 deletion, and Treacher Collins syndromes, and with campomelic dysplasia. In PR-Plus, additional congenital abnormalities accompany the PR triad, but a known syndrome is not indicated. In isolated PR, the triad is the only clinical feature.

That the triad of mandibular dysmorphology, glossoptosis, and CP co-occur is certain. That the onset of these anomalies is a causative sequence is not. Although there is a lack of consensus regarding the etiology of PR, three diagnostic categories exist based on whether mandibular dysmorphology, glossoptosis, and CP appear in isolation or with other anomalies (Figure 1). Syndromic PR is defined when the triad is present as part of a syndrome, appearing coincidentally with Stickler, 22q11.2 deletion, and Treacher Collins syndromes, and with campomelic dysplasia [17,18,34]. PR-Plus is defined when additional congenital abnormalities accompany the PR triad, but a known syndrome is not indicated. Nonsyndromic or isolated PR is defined when the triad is the only clinical feature in an otherwise typically developing infant. It is unknown whether the etiology of PR anomalies varies according to diagnostic category. There are excellent reviews of mandible, tongue, and palate development (e.g., [35,36,37]) and limited studies of mouse models that show PR phenotypes [38,39,40,41], but most studies are descriptive, without a focus on how these anomalies might be mechanistically, molecularly, or developmentally related. 

The idea of PR as a sequence implies that PR phenotypes are developmental consequences of a primary defect. Developmental consequences could occur due to cells sensing and reacting to their physical environment through mechanotransduction, which is the cellular process of translating mechanical forces into biochemical signals or into the activation of diverse signalling pathways [42], or through the differential reaction of specific cell types to a genetic variant. Studies of mechanotransduction have shown that many diseases result from modifications in the force transmissions among cellular components and tissues that can be traced to changes in extra cellular matrix mechanics, cytoskeleton dynamics, the mechanosensing process of the cell, or altered downstream signaling pathways [42,43]. In the case of PR, defects in mechanotransduction of the involved tissues could underlie one or all of the defects, or the genetic variants currently associated with PR-like diseases could be functionally related through a shared genetic network. The lack of a critical study of the molecular and developmental relationships of PR anomalies is at the basis of uncertainty in diagnosis and provides an impetus for future research.

There is no gold standard for diagnosing PR. Diagnosis is rarely made prenatally but can be determined with a physical exam at birth. When diagnosed at birth, PR may be the only malformation noted, or may be associated with other dysmorphic features, with affected infants displaying a wide range of Apgar scores. Syndromic PR patients were found to have significantly lower Apgar scores and longer hospital stays [44]. Even when syndromic PR is diagnosed, there is little to no information available regarding prognosis [25]. Facial anomalies invariably require therapy and close follow-up, and may require corrective surgery, while imposing a financial and emotional burden on patients and their families. Parents of PR individuals bear a particular burden in that the diagnosis is confusing and overwhelming [45] and because of the profound variation in the anomalies, degree of respiratory distress, and eating difficulties [2] that decrease quality of life and cognitive potential.

While most patients can be managed without surgical intervention and many improve with age, a patient may become more symptomatic and the airway obstruction worsened due to the development of conditions such as temporomandibular joint ankylosis [23,46]. Patients presenting with an associated syndrome were more than twice as likely to require surgical intervention than isolated PR cases (53% vs. 25%) [23]. While a tracheostomy involves many quality of life considerations and appears to have a higher mortality associated in syndromic PR patients [47], mandibular distraction osteogenesis (MDO) requires two operations, i.e., one to create mandibular osteomies and apply distraction devices, and a second to remove the devices after completion of distraction and consolidation. Feeding issues may be addressed by glossopexy (tongue–lip adhesion) or MDO [48]. Due to the individuality of each PR case presentation, no one treatment is best suited to all patients, and each possible intervention is accompanied by benefits and risks that must be carefully evaluated by a multidisciplinary team.

## 5. Development of PR Phenotypes

There are three current theories regarding development of PR phenotypes: (1) Mechanical Theory: Mandibular hypoplasia arises between weeks 7 to 11 of gestation, preventing the tongue from descending and interfering with the nasopharynx, causing respiratory and feeding complications [49]; (2) Mandible Compression Theory: Intrauterine compression due to oligo/polyhydramnios is associated with PR phenotype [50]; (3) Neurological Maturation Theory: Fetal oral muscular activity is required for normal development of the mandible. In the absence of normal esophageal motility and pharyngolaryngeal tone due to neurological or muscular defects, mandibular hypoplasia and possible CP are considered secondary defects [51]. Development of the mandibulofacial region involves the first pharyngeal arch and growth and fusion of facial prominences comprised of cells that interact with the neural ectoderm of the forebrain. This requires precise coordination of signaling among diverse cells, tissues, and organs [52,53]. The mesenchymal core of pharyngeal arches is derived from the cranial neural crest and mesoderm and is covered externally by ectoderm-derived epithelium, and internally by endoderm [52]. Early in craniofacial development, the maxillary and mandibular prominences form within the first pharyngeal arch [35,54]. The development of maxillary and mandibular prominences is sensitive to distal-less (*Dlx*) gene dosage, and their distinction within the first pharyngeal arch is achieved by the bounded expression domains of *Dlx5/6* genes that rely on a nested pattern of *Dlx* gene expression [54,55]. Subsequent patterning by a series of transcription factors of various cell populations give rise to part of the upper lip, the maxillae, zygomatic, squamous temporal, and vomer bones from the maxillary prominence, and to Meckel’s cartilage, the mandible, the malleus, incus, and muscles of mastication from the mandibular prominence. Hooper et al. 2017 [56] profiled the transcriptomes of the epithelium and mesenchyme of the various facial prominences at critical periods of murine craniofacial development and revealed dynamic gene expression changes over time [56]. Genes enriched in the maxillary prominence are involved in Wnt, retinoic acid, and Notch signaling pathways, as well as synaptic function, while genes enriched in mandibular prominence are involved in muscle and skeletal development, indicating the transcriptional programs for the formation of the tongue, Meckel’s cartilage, and the mandible [56].

The tongue and mandible have common origins and are coordinated in their development [36]. The anterior 2/3 of the tongue forms from median and lateral tongue buds that arise from the floor of the first pharyngeal arch. These buds grow rostrally and are eventually filled by occipital myoblasts to form the intrinsic tongue muscles. The posterior 1/3 of the tongue is made from swellings originating from the second, third, and fourth pharyngeal arches. Hedgehog, Transforming Growth Factor β (TGFβ), Wnt, and Notch signaling pathways contribute to mediation of appropriate signaling interactions between the epithelial, cranial neural crest, and mesodermal cell populations that are required to form the tongue [57].

**Figure 2:**
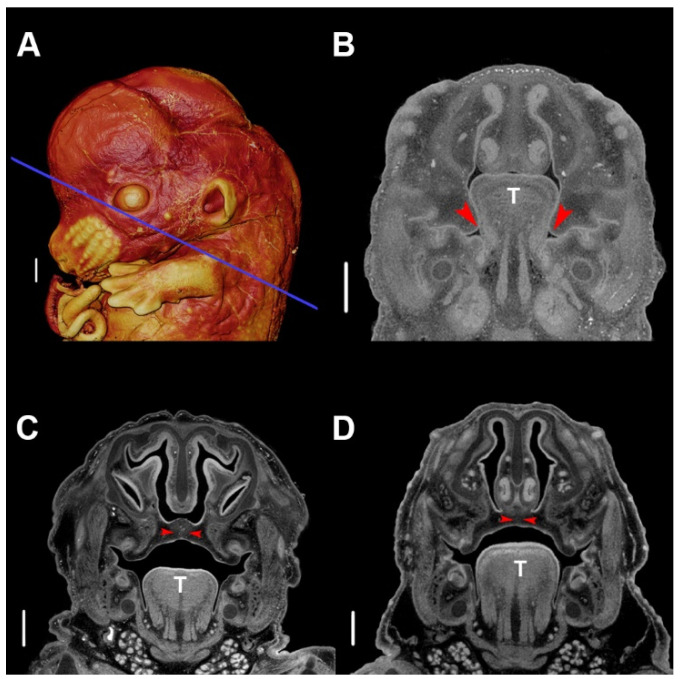
Typical palatogenesis in the murine embryo. (**A**) Three-dimensional (3D) volume-rendering of a phosphotungstic acid (PTA)-enhanced micro-computed tomography (µCT) image of an E13.5 embryo. The blue line indicates the slice plane for all stages. (**B**) Slice image of typical morphology at E13.5, depicting vertical palatal shelves. (**C**) Slice image of typical morphology at E14.5, depicting abutting palatal shelves beginning fusion at the midline. (**D**) Slice of typical morphology at E15.5, depicting fully fused palatal shelves at the midline. The red arrowhead indicates the location of palatal shelves, and T indicates the tongue. Scale bars are 500 µm. Specimens were stained with phosphotungstic acid, as described [57]. µCT scans of PTA stained specimens were acquired by the Center for Quantitative Imaging at The Pennsylvania State University using the 180 kv nanofocus tube of the General Electric v|tom|x L300 nano/microCT system. Image data were reconstructed on a 2024 × 2024 pixel grid as a 32 bit volume, but were reoriented to anatomical planes and reduced to 16 bit volume using Dragonfly 2020.1 (Object Research Systems (ORS) Inc., Montreal, Canada) for image analysis using Avizo 2019.3 (Thermo Fisher Scientific, Waltham, MA, USA). Scan resolution: 5.5 µm.

During mandibulofacial development, medial projections of the maxillary processes form palatal shelves that are initially positioned vertically at E13.5 in mouse (Figure 2A,B). Typically, the developing tongue expands and protrudes relatively high into the oronasal cavity, but subsequently descends into a space provided by the growing mandible. As the tongue descends, the palatal shelves that were restrained by the tongue rotate upward into a horizontal position immediately above the tongue, continue to grow, and eventually begin to fuse around E14.5 (Figure 2C). As the shelves fuse medially at the midline, anteriorly with the primary palate, and superiorly with the nasal septum, the palate separates the nasal and oral cavities, permitting simultaneous respiration and feeding (Figure 2D) [58].

Pathogenesis of PR phenotypes is thought to occur when the tongue is unable to descend into a space diminished by a small and/or malpositioned mandible, preventing the palatal shelves from rotating medially to meet at the midline [59]. This explanation fits logically with gross embryological knowledge of mandibulofacial development and supports a mechanical relationship between the mandible and tongue [35] but there is no consensus on this view [60], it has not been tested experimentally, and a molecular and cellular description of the process is not available. Several human genes required for palatal fusion were identified, and targeted gene mutations in mice revealed many of the molecular determinants of palatal shelf growth, elevation, and fusion [61]. As noted above, many of the genes involved in tongue development were identified [55], and gene expression patterns of early mandibular development are known [53,54]. What is not known is how these genetic instructions, or a totally different set, are integrated in the pathogenesis of PR to produce the triad of phenotypes.

**Figure 3:**
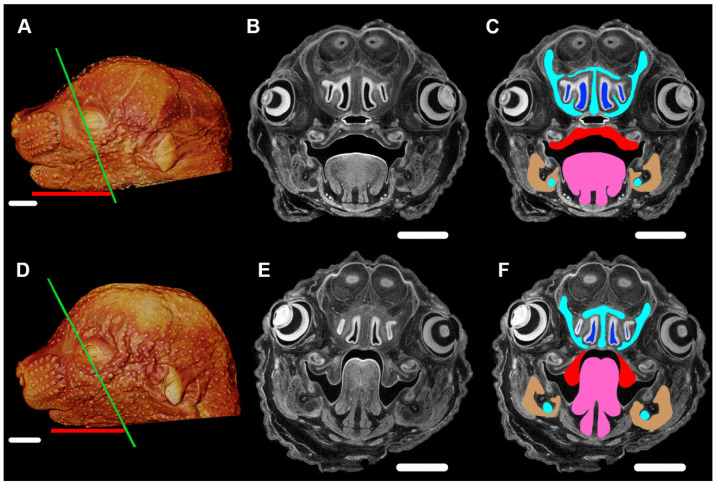
Pierre Robin phenotype of mandible, tongue, and cleft palate in the *Prdm16^cGT/cGT^* embryos visualized by PTA-enhanced µCT. (**A**) Three-dimensional volume rendering of a PTA-enhanced µCT image of an E15.5 *Prdm16^+/+^* mouse showing craniofacial morphology of a typically developing embryo. (**B**) Slice image of typical morphology, plane of section indicated by the green line in (**A**,**C**). Same image as (**B**), highlighting critical tissues of the PR-CP phenotype, namely, the nasal capsule and Meckel’s cartilage (aqua), nasal airways (blue), palatal shelves (red), tongue (pink), and mandible (tan). (**D**) 3D volume rendering of a PTA-enhanced *Prdm16^cGT/cGT^* mouse showing the PR-CP phenotype. (**E**) Slice image of PR-CP morphology, plane of section indicated by the green line in (**D**,**F**). Same image as (**E**), highlighting critical tissues of the PR-CP phenotype, namely, the nasal capsule and Meckel’s cartilage (aqua), nasal airways (blue), palatal shelves (red), tongue (pink), and mandible (tan). (**A**,**D**) Length of mandible shown by red bar. Scale bars in (**A**,**D**) are 1 mm. Scale bars in (**B**,**C**,**E**,**F**) are 500 µm. Imaging processing as described for Figure 2.

An example of PR phenotypes being produced by changes in a single protein coding gene is now available in a mouse model. *Prdm16* (PR/SET Domain 16) encodes a transcriptional cofactor that regulates TGFβ signaling, with expression patterns that are consistent with a role in palate and craniofacial development [38]. Nonsyndromic CP caused by an intronic *Prdm16* splicing mutation in the cleft secondary palate 1 (*csp1*) *N*-ethyl-*N*-nitrosurea-induced mouse model was thought to be the result of micrognathia and failed palate shelf elevation due to physical obstruction by the tongue, resembling human PR-like cleft secondary palate [38]. Conditional gene trap cassettes were used to develop a generic strategy for generating conditional mutations, validated in mice carrying a multipurpose allele of the *Prdm16* transcription factor [39]. The phenotype of the *Prdm16^cGT^* and *Prdm16^cGTreinv^* mice was virtually identical to the previously reported *Prdm16^csp1^* phenotype [38,39]. By E15.5, *Prdm16^+/+^* embryos showed normal anatomy of the mandible, tongue, and palate (Figure 3A–C) while *Prdm16^cGT/cGT^* embryos showed the PR-CP phenotype consisting of a tongue protruding upward against cartilage of the developing cranial base, a CP, narrowed airways, and a hypomorphic mandible (Figure 3D–F).

CP can occur with apparently normal tongue and mandible development, but mutations affecting early mandibular development can have deleterious effects on tongue formation and subsequently result in CP. Using an in vitro suspension palate culture system, a primary role for *Prdm16* in the developing mandible or tongue and not the palate shelves is evident in *Prdm16^csp1^* mutants that undergo normal palate elevation and fusion upon removal of the mandible and tongue [38]. Similarly, a mutation of *Erk2* in neural crest derivatives phenocopies the human PR phenotype, and highlights the interconnection of palate, tongue and mandible development [62]. *Wnt1-Cre;Erk2^fl/fl^* mice exhibited CP with elevation defects, microglossia, tongue malposition, disruption of the tongue muscle patterning, and compromised tendon development [62]. Culturing these mutants in the absence of the tongue and palate was sufficient to rescue the clefting defects, supporting a primary malformation of the mandible and/or tongue as the cause of impaired palate shelf elevation. The tongue phenotype was rescued after culture in isolation, however, indicating that it might also be a secondary defect [62]. The consensus view is that influences from other craniofacial and oral structures, including movement of the tongue and growth of the cranial base and mandible contribute to palatal shelf elevation and fusion, but intrinsic properties of the palatal shelves also play a role [61]. A recent study of primary palate fusion demonstrated the unique expression profiles of each cell population involved, how gene expression information for single cells representing these cell populations are impacted by mutations or environmental insults, and how signals that integrate the behavior of these cell populations are required during fusion [63].

A thorough understanding of the production of PR phenotypes requires knowledge of the molecular pathways that might contribute to the regulation of processes that supervise development of the tongue, palate, and mandible individually, as well as the hierarchical or nested control of the integration of these structures. The biomechanical forces produced and sensed by tissues of varying material properties as they expand with growth certainly contributes to mandibulofacial development, and so, logically, should play a role in the production of PR phenotypes. Determining the role of these forces requires a serious study of how mechanical signals are transformed into biological signals (mechanotransduction) during mandibulofacial development.

## 6. Genetics of PR

PR is poorly characterized at the genetic level. The transcription factor SOX9 is a master regulator of chondrocyte fate essential for cartilage formation and skeletal development. Intragenic, loss-of-function *SOX9* mutations cause campomelic dysplasia, of which PR is a feature [64,65]. Variants affecting the spatiotemporal activity of *SOX9* regulatory elements cause isolated PR [66], and regulatory *SOX9* variants were also identified in PR-Plus [34,67,68]. *SOX9* positively regulates transcription of *Col2a1*, *Col11a1*, and *Col11a2* during cartilage formation in mouse and chicken [69,70,71]. Mutations in these three genes cause Stickler syndrome, the syndrome most commonly associated with PR [27,31,72]. The involvement of these genes in PR underscores the importance of the proper formation of Meckel’s cartilage to mandibular outgrowth, perturbation of which can be a primary event in PR. However, PR occurs in PR-Plus forms and in association with a wide variety of less common syndromes, for which genetic causes are not completely known [73,74,75]. Knowledge of these genes may give insight into the wider morphogenetic impact of their variants or mutations and thereby influence prediction of clinical trajectories, leading to improved, patient-specific treatments. Table 1 lists select genes for human syndromes associated with PR phenotypes as reported in the Online Mendelian Inheritance in Man (OMIM; www.omim.org), the Monarch Initiative (www.monarchinitiative.org), and reviewed in Tan et al. 2013 [74] and Logjes et al. 2018 [73]. The variety of genes listed in Table 1 and these databases and reviews reveal the genetic and mechanistic complexity of PR. Previous screens looked for intragenic mutations in SOX9 and other candidate genes in syndromic PR [76], but no real concerted effort for nonsyndromic PR in humans. Further investigation is required to identify and confirm that genes implicated in human PR are causative through animal models.

## 7. Animal Models as a Means for Understanding PR

There are many animal models exhibiting PR-related phenotypes, including mandibular dysmorphology, malformed tongue, and/or CP (Mouse Genome Informatics, the Monarch Initiative, [73,74]) (Table 2). The various candidate genes involved in these models have diverse functions, reflecting the heterogeneity of genetic influences that can result in a PR phenotype. Heterozygous inactivation of *Sox9* results in a shortened mandible, abnormal tongue, and CP [77]. Conditional, heterozygous deletion of *Sox9* in the neural crest also results in a shortened mandible and CP [41,78]. One model involves deletion of a long-range enhancer element that regulates *Sox9* expression in mice and is conserved in humans in the region affected by deletions and translocations in some PR-Plus cases [41]; however, it does not display the full PR triad, lacking tongue and palate defects. Loss-of-function mutations in collagen genes were found in syndromes associated with PR phenotypes and mice homozygous for chondrodysplasia (*Col11a1^cho/cho^*), cartilage matrix deficiency (*Acan^cmd/cmd^*), and disproportionate micromelia (*Col2a1^Dmm/Dmm^*) exhibited macroglossia and tongue obstruction during palatogenesis resulting in CP, thereby supporting the hypothesis for the PR sequence [79,80]. TGFβ/ 

Bone Morphogenetic Protein (BMP) signaling is critical for the development of the mandible, the palate and the tongue [57,81,82]. PR-related phenotypes are observed in the null or conditional knockout mice of the genes in TGFβ/BMP signaling, including *Acvr2a* [83], *Acvr1* [84], *Bmp2* [85], *Bmp7* [86], *Prdm16* [38], and *Tak1* [87], indicating a potential role of TGFβ/BMP signaling in PR pathogenesis.

While studies of animal models provided candidate genes for PR and insights into the underlying pathogenic molecular pathways, they did not elucidate whether physical constraints contribute to abnormal development, or to what extent phenotypes represent a causative series stemming from a primary event, such as micrognathia. For example, mandibulofacial dysostoses, such as Treacher Collins syndrome (caused by mutations in *TCOF1*, *POLR1C*, *POLR1D*), Miller syndrome (caused by mutations in *DHODH*), and Nager syndrome (caused by mutations in *SF3B4*), were reported to include features of PR in patients, but may not represent true PR phenotypes. Studies of a Treacher Collins mouse model showed that the mandibulofacial dysostosis is due to abnormalities in ribosomal biogenesis and increased apoptosis, but did not demonstrate the PR phenotype of glossoptosis leading to CP [88,89]. Another instance that questions whether constraint contributes to PR phenotype is the neural crest cell-specific mutant line, *Med23^fl/fl^*;*Wnt1-Cre*, generated by Dash et al. 2020 [90] that exhibits micrognathia, glossoptosis, CP and cleidocranial dysplasia, providing a novel PR mouse model. To examine the role of the tongue in CP in this model, the maxillary apparatus of unfused palates in mutant and control E13.5 embryos were dissected and placed in ex vivo culture. After 72 h of culture, the control palatal shelves developed rugae and fused, while the palatal shelves of mutant embryos formed rugae but remained unfused. These necessary and informative assays revealed the enduring inability of the *Med23^fl/fl^;Wnt1-Cre* palatal shelves to close when an obstructive tongue is no longer present, but can not account for the potential developmental effects of a large, superiorly placed tongue during palatal shelf formation.

Although animal models were successfully used to reveal the developmental and pathogenic mechanisms in the mandible, tongue, and/or palate, most of the candidate genes identified from animal models are not confirmed in PR patients. Furthermore, new models must be established to study the PR-associated mutations found in patients with PR and other related syndromes. Novel animal models for PR could help us better understand the pathogenic mechanisms and facilitate discovering diagnostic strategies and therapeutic solutions for PR.

## 8. Conclusions

The etiology of PR remains unclear despite recent advances in craniofacial research. While the primary defect in many PR patients appears to be mandibular hypoplasia, as we learn more about the complex relationship among developing mandibulofacial structures the developmental basis of the condition may be variable and is not yet clearly elucidated. The lack of information regarding the etiology of PR phenotypes motivates novel experimental study of these conditions. Mouse models of the PR phenotype, such as the *Prdm16* gene trap model shown in Figure 2, provide a means for investigating the role of mechanotransduction, the molecular basis, and the phenotypic consequences of normal and perturbed development, and could allow further definition of the mechanisms underlying development of the PR phenotype. 

## Figures and Tables

**Table 1:** Select genes associated with human PR phenotypes.

Gene Symbol	Gene Name	Syndrome(s)	MIM Phenotype Number
*AMER1*	Apc membrane recruitment protein 1	Osteopathia striata with cranial sclerosis	300373
*AP3D1*	Adaptor related protein complex 3 subunit delta 1	Hermansky–Pudlak syndrome 10	617050
*BMP2*	Bone morphogenetic protein 2	Short stature, facial dysmorphism, and skeletal anomalies with or without cardiac anomalies	617877
*COG1*	Component of oligomeric golgi complex 1	Congenital disorder of glycosylation, type IIg	611209
*COL11A1*	Collagen, type XI, alpha-1	Stickler syndrome, type II; Marshall syndrome	604841; 154780
*COL11A2*	Collagen, type XI, alpha-2	Otospondylomegaepiphyseal dysplasia, autosomal dominant; Otospondylomegaepiphyseal dysplasia, autosomal recessive	184840; 215150
*COL2A1*	Collagen, type II, alpha-1	Stickler syndrome, type I	108300
*DHODH*	Dihydroorotate dehydrogenase	Miller syndrome	263750
*EDN1*	Endothelin 1	Auriculocondylar syndrome 3	615706
*EFTUD2*	Elongation factor Tu guanosine triphosphate binding domain containing 2	Mandibulofacial dysostosis, Guion–Almeida type	610536
*EIF4A3*	Eukaryotic translation initiation factor 4a3	Robin sequence with cleft mandible and limb anomalies	268305
*MAP3K7*	Mitogen-activated protein kinase kinase kinase 7	Frontometaphyseal dysplasia 2	617137
*MYMK*	Myomaker, myoblast fusion factor	Carey–Fineman–Ziter syndrome	254940
*PDHA1*	Pyruvate dehydrogenase E1 subunit alpha 1	Pyruvate dehydrogenase E1-alpha deficiency	312170
*PGAP3*	Post-glycophosphatidylinositol attachment to proteins phospholipase 3	Hyperphosphatasia with mental retardation syndrome 4	615716
*PGM1*	Phosphoglucomutase 1	Congenital disorder of glycosylation, type It	614921
*PIGA*	Phosphatidylinositol glycan anchor biosynthesis class A	Multiple congenital anomalies–hypotonia–seizures syndrome 2	300868
*POLR1C*	RNA polymerase I and III subunit C	Treacher Collins syndrome 3	248390
*POLR1D*	RNA polymerase I and III subunit D	Treacher Collins syndrome 2	613717
*RBM10*	RNA-binding motif protein 10	TARP syndrome	311900
*SATB2*	Special AT-rich sequence-binding protein 2	Glass syndrome	612313
*SLC10A7*	Solute carrier family 10 member 7	Short stature, amelogenesis imperfecta, and skeletal dysplasia with scoliosis	618363
*SLC26A2*	Solute carrier family 26 member 2	Diastrophic dysplasia	222600
*SNRPB*	Small nuclear ribonucleoprotein polypeptides B and B1	Cerebrocostomandibular syndrome	117650
*SOX9*	Sry-box 9	Campomelic dysplasia	114290
*SF3B4*	Splicing factor 3b subunit 4	Nager syndrome	154400
*TBX1*	T-box transcription factor 1	Velocardiofacial syndrome	192430
*TCOF1*	Treacle ribosome biogenesis factor 1	Treacher Collins syndrome 1	154500
*TGDS*	Thymidine diphosphate-glucose 4,6-dehydratase	Catel–Manzke syndrome	616145

**Table 2:** Select animal models for PR phenotypes.

Animal Model	Species	Gene	Mutation	Phenotypes				References
				Jaw	Tongue	Palate	Others	
*Acan^cmd/cmd^*	Mouse	*Acan*	Intragenic deletion in *Acan*	Micrognathia or agnathia	Underdeveloped	Cleft palate	Short-limbed chondrodystrophy	[80,91]
*Acvr2a^tm1Zuk^*	Mouse	*Acvr2a*	*Acvr2a* null	Micrognathia, defects in Meckel′s cartilage	None reported	Cleft palate	None reported	[83]
*Acvr1^fl/fl^*; *Wnt1-Cre*	Mouse	*Acvr1*	*Wnt1-Cre* conditional knockout of *Acvr1*	Micrognathia	None reported	Cleft palate	Enlarged frontal fontanels, incomplete zygomatic arches, squamosal bones lack the retrotympanic process; smaller temporal squama	[84]
*Bmp2^fl/fl^*; *Wnt1-Cre;R26R^mTmG^*	Mouse	*Bmp2*	*Wnt1-Cre* conditional knockout of *Bmp2*	Micrognathia	Malformed tongue	Cleft palate	A reduced size of craniofacial bones	[85]
*Bmp7^Δ/Δ^*	Mouse	*Bmp7*	*Bmp7* null	Impaired Meckel’s cartilage development; lack of a mandibular symphysis and mandibular mental spine formation	Misplaced origin of genioglossus muscle	Cleft palate	Alteration of oral cavity morphology	[86,92]
*Col11a1^cho/cho^*	Mouse	*Col11a1*	Intragenic deletion in *Col11a1*	Micrognathia or agnathia	Underdeveloped	Cleft palate	Short-limbed chondrodystrophy	[80,93]
*Col2a1^Dmm^*	Mouse	*Col2a1*	Disproportionate micromelia (Dmm) semi-dominant mutation	Mandibular growth retardation, coupled with relative macroglossia in E14	Relative tongue size to Meckel’s cartilage length significantly greater at E14.75 compared to control	Cleft palate	Mild dwarfism three weeks after birth in heterozygotes	[79]
*Edn1^−/−^*	Mouse	*Edn1*	*Edn1* null	Short and deformed mandibular bones	Most of tongue missing	Cleft palate	Thin anterior neck and hypoplastic auricles, aberrant zygomatic andtemporal bones, absent auditory ossicles and tympanic ring	[74,94]
*Egfr^−/−^*	Mouse	*Egfr*	Targeted intragenic deletion in *Egfr*	Under-developed lower jaw	None reported	Cleft palate	Narrow, elongated snouts	[95]
*Erk2^fl/fl^*; *Wnt1-Cre*	Mouse	*Erk2*	*Wnt1-Cre* conditional knockout of *Erk2*	Micrognathia and mandibular asymmetry	Malformed tongue	Cleft palate, failed palate elevation	None reported	[62]
*pMes-Fgf10*; *Wnt1-Cre*	Mouse	*Fgf10*	*Wnt1-Cre* conditional transgene of *Fgf10*	None reported	Heightened tongue	Failed palate elevation	None reported	[87]
*Hoxa2D1*	Mouse	*Hoxa2*	*Hoxa2* null	Duplicated Meckel′s cartilage	None reported	Cleft palate	External ear defects, duplication of the ossification centers of the bones of the middle ear	[96]
*Med23^fl/fl^*; *Wnt1-Cre*	Mouse	*Med23*	*Wnt1-Cre* conditional knockout of *Med23*	Micrognathia, hypoplastic Meckel’s cartilage	Glossoptosis	Cleft palate	Cleidocranial dysplasia: Agenesis of nasal cartilage and bones, abnormal development of the tympanic ring and skull bones	[90,97]
*Msx1^−/−^*	Mouse	*Msx1*	*Msx1* null	Shortened mandible and maxilla	None reported	Cleft palate	Failure of tooth induction; Abnormalities of the nasal, frontal and parietal bones, and of the malleus in the middle ear; cyanosis	[98]
*Prdm16^cGT^*	Mouse	*Prdm16*	*Prdm16* null	Micrognathia, smaller Meckel′s cartilage	Abnormal positioning and morphology of the tongue	Cleft palate	Respiratory failure and abdominal distention, reduced ossification of the frontal and parietal bones, nasal cartilage appears shortened, abnormal retinal folds; hypoplasia of choroid plexi, salivary glands, lungs, cardiac ventricules	[39]
*Prdm16^csp1^*	Mouse	*Prdm16*	Intronic splice mutation in *Prdm16*	Micrognathia, smaller Meckel′s cartilage	Abnormal positioning and morphology of the tongue	Cleft palate	Respiratory failure and abdominal distention, reduced ossification of the frontal and parietal bones, nasal cartilage appears shortened, abnormal retinal folds; hypoplasia of choroid plexi, salivary glands, lungs, cardiac ventricules	[38]
*Ptprs^−/−^; Ptprf^−/−^*	Mouse	*Ptprs*, *Ptprf*	*Ptprs;Ptprf* double-knockout	Micrognathia	Microglossia/glossoptosis	Cleft palate	Dysmorphic cranial bone and cartilage	[99]
*Satb2^tm1(cre)Vit^*	Mouse	*Satb2*	*Satb2* null	Micrognathia	Microglossia	Cleft palate	Microcephaly, nasocapsular and premaxillary hypoplasia; fully penetrant incisor adontia	[100]
*Snai1/2-dko*	Mouse	*Snai1/Snai2*	Neural-crest-specific *Snai1* deletion on a *Snai2^−/−^* genetic back-ground	Micrognathia, fused mandible and a failure of Meckel′s cartilage to extend the mandible	None reported	Cleft palate	Enlarged parietal foramen in skull vault	[101]
*Sox9^+/−^*	Mouse	*Sox9*	Heterozygous knockout of *Sox9*	Micrognathia	Bifurcated tongue	Cleft palate	Hypoplasia of cartilaginous skeletal elements	[77]
*Sox9^fl/+^*; *Wnt1-Cre*	Mouse	*Sox9*	Heterozygous *Wnt1-Cre* conditional knockout of *Sox9*	Micrognathia	None reported	Cleft palate	Mildly hypoplastic craniofacial skeleton	[78]
*Sox9^fl/+^*; *Wnt1-Cre2*	Mouse	*Sox9*	Heterozygous *Wnt1-Cre* conditional knockout of *Sox9*	Micrognathia	None reported	Cleft palate in 50% of mutant embryos	None reported	[41]
*Sox9 mEC1.45del/del*	Mouse	*Sox9*	Knockout of *Sox9* enhancer mEC1.45	Altered mandibular morphology	None reported	None reported	Reduction in weight gain	[41]
*Sox11^fl/fl^*; *EIIa-Cre*	Mouse	*Sox11*	*Sox11* null	Micrognathia	Displaced tongue position	Cleft palate with retardation to palatal shelf elevation	None reported	[102]
*Tak1^fl/fl^*; *Wnt1-Cre*	Mouse	*Tak1*	*Wnt1-Cre* conditional knockout of *Tak1*	Micrognathia	Malformed tongue	Cleft palate	Hypoplastic calvarial bones	[87]
*Tbx1^−/−^*	Mouse	*Tbx1*	*Tbx1* null	Micrognathia	None reported	Cleft palate	Hypoplasia of the thymus and parathyroid glands, cardiac outflow tract abnormalities, abnormal facial structures, abnormal vertebrae	[103]
*Tcof1^+/−^*	Mouse	*Tcof1*	Heterozygous knockout of *Tcof1*	Micrognathia/retrognathia	None reported	Cleft palate	Agenesis of the nasal passages, abnormal maxilla, exencephaly, anophthalmia	[88,104]
*Tgds^bub^/Tgds^bub^*	Mouse	*Tgds*	N-ethyl-N-nitrosourea-induced mutation	Micrognathia	None reported	Cleft palate	None reported	[105]
hpmd-line 171a	Mouse	Unknown	N-ethyl-N-nitrosou-rea-induced mutation	Hypoplastic mandible	None reported	Cleft palate	Split in xyphoid process, malformation of first brachial arch derivatives	[76,106]
A/WySn	Mouse	Unknown	Unknown	Retrognathia	None reported	Cleft palate	None reported	[107]
CP1 NSDTR	Dog	*DLX6*	A long interspersed nuclear element-1 insertion in *DLX6*	Relative micrognathia	None reported	Cleft palate	None reported	[108]
*crispld2^KD^*	Zebrafish	*crispld2*	Morpholino knockdown of *crispld2*	Loss of lower jaw structures	None reported	Malformations of the palate	Truncated body, shortened and curved tail with cardiac edema, clefting of the ethmoid plate	[109]
*faf1^KD^*	Zebrafish	*faf1*	Morpholino knockdown of *faf1*	Under-developed jaw	None reported	None reported	Smaller head; “open-mouth” phenotype	[110]
*polr1c^−/−^*	Zebrafish	*polr1c*	*polr1c* knockout (*polr1c^hi1124Tg^*) generated by insertion mutagenesis	Hypoplastic mandible	None reported	Cleft palate, smaller ethmoid plate	Smaller heads, microphthalmia, pericardial edema	[111,112]
*polr1d^−/−^*	Zebrafish	*polr1d*	*polr1d* knockout *(polr1d^hi2393Tg^*) generated by insertion mutagenesis	Hypoplastic mandible	None reported	Smaller ethmoid plate	Smaller heads, microphthalmia, pericardial edema	[111]
*tcof1^KD^*	Zebrafish	*tcof1*	Morpholino knockdown of *tcof1*	Hypoplastic mandible	None reported	Smaller and dysmorphic ethmoid plate	Cranioskeletal hypoplasia in the frontal, premaxillary, and maxillary elements	[113]
